# Investigation of synergistic mechanism and identification of interaction site of aldose reductase with the combination of gigantol and syringic acid for prevention of diabetic cataract

**DOI:** 10.1186/s12906-016-1251-5

**Published:** 2016-08-12

**Authors:** Jie Wu, Xue Li, Hua Fang, Yanqun Yi, Dan Chen, Yan Long, Xinxin Gao, Xiaoyong Wei, C-Y. Oliver Chen

**Affiliations:** 1 Department of Histology and Embryology, Guangzhou University of Chinese Medicine, 510006 Guangzhou, China; 2 Antioxidants Research Laboratory, Jean Mayer USDA Human Nutrition Research Center on Aging, Tufts University, 02111 Boston, MA USA

**Keywords:** Synergism, Diabetic cataract, Site-directed mutagenesis, Gigantol, Syringic acid, Aldose reductase

## Abstract

**Background:**

Gigantol and syringic acid (SA) have been shown to synergistically prevent formation of diabetic cataract (DC). However, the exact mechanism of this effect is unknown. Here, we investigate the effect of these compounds on the activity of aldose reductase (AR) and cataract formation.

**Methods:**

We examined the synergistic anti-cataract efficacy of gigantol and SA in the high glucose- and streptozotocin -induced DC rat model; synergism was evaluated using Jin’s formula. We investigated possible mechanisms of action by measuring AR expression and activity and levels of sorbitol using enzyme kinetics, Western blot, and RT-PCR. Finally, we examined binding interaction between AR and both compounds using a combination of site-directed mutagenesis, recombinant expression of wild-type and mutant proteins, and enzyme kinetics.

**Results:**

Combination treatment of gigantol and SA synergistically protected both HLECs(human lens epithelial cells) grown in vitro and DC formation in STZ-induced rats in vivo. Synergism was attributed to inhibition of AR activity, downregulation of AR expression via impaired transcription, and decreased sorbitol levels. Enzyme kinetics studies showed that the activity of an AR Asn160Ala mutant protein was significantly decreased compared to wild-type AR, confirming that Asn160 is a key residue for interaction between AR and both compounds.

**Conclusion:**

Combined administration of gigantol and SA synergize to enhance anti-cataract efficacy. The synergistic effect is mainly attributed to disruption of the polyol pathway and inhibition of AR activity.

## Background

Cataract formation in patients suffering from diabetes is a major cause of blindness. This is particularly true in developed countries in which individuals tend to eat high-fat diets, which are linked to an increased incidence of diabetes. Cataract formation associated with diabetes (diabetic cataract, DC) can severely affect patients’ vision and quality of life [1]. Importantly, cataracts account for most cases of blindness in diabetic patients [2, 3].

Although cataract surgery is quite common and provides an effective cure, a better understanding of cataract development might help delay or even prevent cataract onset in diabetic patients. Furthermore, patients with diabetes mellitus have more complications associated with cataract surgery, including rapid acceleration of retinopathy, rubeosis, macular edema, and cystoid macular edema [4–7]. Both diabetes and cataracts pose an enormous health and economic burden, particularly in developing countries, where diabetes treatment is insufficient and cataract surgery is often inaccessible [4, 8]. Thus, there is an urgent need for inexpensive, non-surgical approaches for cataract treatment [9]. Importantly, a 10-year delay in cataract onset is predicted to cut the number of necessary cataract extractions to half [10]. Thus, this necessitates the search for alternative pharmacological measures to treat this disorder [11].

The pathogenesis of diabetic cataract development is still not fully understood. Multiple mechanisms, including increased sorbitol pathway activity [12, 13], non-enzymatic glycation and glycoxidation [14, 15], enhanced oxidative-nitrosative stress [16, 17], protein kinase C signaling [18, 19], poly (ADP-ribose) polymerase (PARP) activity [20, 21], and lipoxygenase activation [22, 23] have all been implicated in the pathogenesis of chronic diabetic complications [24].

Previous studies have established that the enzyme aldose reductase (AR) catalyzes the reduction of glucose to sorbitol through the polyol pathway, a process linked to the development of diabetic cataracts [4]. AR is the first and rate-limiting enzyme of the polyol pathway [25]. Polyols, such as sorbitol, physiologically accumulate inside cells. Under diabetic conditions, AR promotes the conversion of excess glucose to sorbitol in tissues [26]. The accumulation of sorbitol in lens fiber cells leads to an increase in lens osmotic stress [27]. AR-dependent synthesis of excess polyols has been implicated as a primary mechanism leading to diabetic cataracts [28]. As such, inhibition of AR signaling in the lens could represent a useful strategy for diabetic cataract prevention. Along these lines, a variety of AR inhibitors (ARIs) have been shown to effectively inhibit AR activity and decrease the risk associated with diabetes mellitus; moreover, these inhibitors have also been shown to mitigate polyalcohol metabolism-related pathogenesis in diabetic patients [29].

Caulis Dendrobii, a traditional Chinese herb, is the fresh or dry stem of the *Dendrobium* Sw. plants of *Orchidaceae*. According to several Chinese medical reports, Caulis Dendrobii has been shown to improve vision [30, 31]. We have previously extracted gigantol and syringic acid (SA) from Dendrobii and have shown that either compound alone can significantly inhibit AR activity and help prevent DC formation in rats [32, 33]. As a continuation of our efforts directed towards the development of diabetes-associated anti-cataract agents derived from natural sources, we found that the combination of gigantol and SA protects against DC formation better than either agent alone. Molecular docking analysis predicts that binding between AR and the combination of gigantol and SA occurs via amino acid residue Asn160 within AR [34]. We have previously created eye drops containing gigantol and SA, and preclinical studies have shown that these drops are non-toxic with little to no irritation in several animal models. Thus, these eye drops were deemed safe and determined to have several drug-like properties [35]. Taken together, eye drops containing gigantol and SA are worth further examination as a potential anti-cataract therapeutic.

The molecular mechanisms underlying the synergistic effect of gigantol and SA are not fully understood. Moreover, many of the previous studies have made use of galactose- or hydrogen peroxide-induced cataract animal models, which typically fail to mimic human disease [28, 36]. As such, in this study, we examined the effect of gigantol and SA combination in streptozotocin (STZ)-induced diabetes in vivo and high glucose-induced diabetes in vitro. Cell viability was analyzed by MTT (3-(4, 5-dimethylthiazol-2-yl)-2, 5-diphenyltetrazolium bromide) assay, and the synergistic effects were evaluated by applying Jin’s formula [37, 38]. AR activity and expression, sorbitol levels, and binding between AR and both compounds were examined to better understand the potential synergistic mechanisms governing the anti-cataract properties of these agents.

## Methods

### Materials and preparation of eye drops

STZ, MTT, DMSO (dimethyl sulfoxide, and DL-glyceraldehyde were purchased from Sigma America Corp. NADPH (reduced form of nicotinamide-adenine dinucleotide phosphate) was obtained from Italian Roth Corp. Pirenoxine eye drops were obtained and produced by Wuhan Tiantianming Pharmacy Co. Ltd. (P. R. China). Ammonium acetate was purchase from Gene-Tech Co. Ltd. All other reagents were obtained domestically and were deemed to be analytically pure.

Live specimens of *Dendrobium aurantiacum* var.*denneanum* (kerr) Z.H. Tsi were acquired from Wan’an Dendrobium Industry and Development Co., Ltd. (Sichuan, P.R. China). These specimens were authenticated by Professor Tingmo Zhang of the Chengdu University of Chinese Medicine. Gigantol and SA were extracted at >98 % purity using the method previously described [39, 40].

The eye drops containing both gigantol and syringic acid were prepared as previously described [35]. Briefly, gigantol (1 g), SA (1.25 g), and ethylparaben (0.3 g) were added to 1 L of buffer solution (940 mL of 12.4 g/L boric acid buffer and 60 mL of 19.1 g/L borax buffer), followed by boiling for 15 min. After the mix was cooled and the pH was adjusted to 7.0 using the boric acid buffer, the resulting solution was added with 2.2 g sodium chloride and then filtered using a 0.22 *μ*m membrane to generate the drops. Eye drops containing gigantol or SA alone were prepared as described above except for the addition either gigantol alone (2 g) or SA alone (2.5 g).

### Cell culture, treatment, and cell viability assay

HLECs (human lens epithelial cells) line SRA01/04, generously provided by the Ophthalmology Center of the Sun Yat-sen University (P.R. China), was cultured in Dulbecco’s modified Eagle’s medium (DMEM; with 5.56 mmol/L glucose) supplemented with 20 % fetal bovine serum (FBS), 100 IU/mL penicillin, and 100 mg/mL streptomycin at 37 °C in a 5 % CO_2_ humidified atmosphere [41]. HLECs were harvested at confluency by trypsinization, and fresh culture medium was added to generate single cell suspensions for use in cell viability assays. HLECs were then seeded in 96-well cell culture grade microplates at a density of 1 × 10^5^ cells/well in 100 μL for 24 h followed by incubation with high glucose (50 mmol/L glucose-DMEM; model group), normal glucose (5.56 mmol/L glucose-DMEM; normal control group), or mannitol (5.56 mmol/L glucose–DMEM with 45 mmol/L mannitol; osmolarity control group) [42].

Cells were treated for 72 h with high glucose (50 mmol/L glucose-DMEM) and various concentrations of gigantol and syringic acid, either alone or in combination. Gigantol was administered at doses of 0, 0.1, 0.5, 1.0, and 2 μg/mL. Syringic acid was administered at doses of 0, 0.125, 0.625, 1.25, and 2.5 μg/mL. The doses used for the combination are listed in Table 1. Cell viability was assessed by MTT assay. After the indicated treatments, 10 μL of 5 mg/mL MTT reagent was added to each well of a 96-well microplate and incubated in the dark at 37 °C for 4 h. Finally, 200 μL DMSO was added as the MTT formazan product solvent to each well with vigorous mixing after the supernatant was removed. Afterwards, the optical density (OD) at 570 nm was measured with an EnSpire™ Multimode Plate Reader. Cell viability was calculated from the absorbance ratios in the control group and the sample group. Morphological changes of cells in each group were observed under an inverted microscope (Olympus, Japan).

**Table 1:** The cell viability (%) of HLECs after treatment with gigantol, syringic acid and their combination in the presence of high glucose

gigantol (μg/mL)	SA(μg/mL)				
	0	0.125	0.625	1.25	2.5
0	28.23 ± 1.12^*^	32.62 ± 1.56	36.21 ± 2.18	41.52 ± 1.53	47.72 ± 1.49
0.1	35.33 ± 1.87	68.02 ± 1.27	69.01 ± 1.79	71.09 ± 1.94	73.99 ± 1.35
0.5	39.55 ± 1.25	69.77 ± 2.22	75.05 ± 2.15	76.26 ± 1.28	78.96 ± 1.57
1	44.71 ± 2.51	71.16 ± 1.61	73.66 ± 1.24	82.51 ± 1.53	83.02 ± 1.82
2	50.65 ± 1.47	72.76 ± 1.59	74.13 ± 1.27	83.43 ± 1.56	89.16 ± 1.32

Cells were incubated with various concentrations of gigantol, SA and their combination in presence of high glucose (50 mM, containing medium) for 72 h. The cell viability of normal group (5.56 mM glucose), osmolarity control group and model group were 100 ± 0.89 %, 98.33 ± 0.24 % and 28.23 ± 1.12 %, respectively. Cells were treated for 72 h, respectively. independent experiments. Data were expressed as mean ± SD (*n* = 3), ^*^ *P* < 0.01 vs all other groups

### Evaluation of synergism

The synergistic effect of gigantol and syringic acid was analyzed by applying the modified Bürgi formula (i.e., Jin equation) [37, 38]. The formula is q = EA + B/ (EA + EB − EA × EB), where EA + B, EA, and EB are the average effects of the combination treatment, gigantol alone, and SA alone, respectively. The q value < 0.85, 0.85-1.15, and ≥ 1.15 indicate antagonism, additive effects, and synergism, respectively.

### AR activity assay in HLECs

AR activity in the cytosolic fraction of HLECs was spectrophotometrically estimated as previously described [42–46]. Harvested HLECs were first ultrasonically disrupted in 100 mmol/L PBS (phosphate buffer saline, pH 7.4), followed by centrifugation at 2000 × *g* for 10 min. The reaction was carried out in 1.5 mL incubation medium (100 mmol/L PBS, pH 6.8, 0.1 mmol/L *DL*-glyceraldehyde, 0.15 mmol/L NADPH, and 100 μL AR enzyme fraction); *DL*-glyceraldehyde was added the last. Enzyme activity was measured spectrophotometrically by estimating NADPH oxidation from a decrease in absorbance at 340 nm. The assay was carried out at room temperature with an appropriate blank subtracted from each reaction to correct for non-specific oxidation of NADPH during the measurement. One unit of enzyme activity is defined as the amount of enzyme catalyzing the oxidation of 1 μmol NADPH/min under the present assay conditions.

### Effect of combined gigantol and SA on STZ-induced DC lens opacification *in vivo*

#### Animal handling and care

Totally 100 Wistar rats with an average body weight of 220 ± 10 g (aged 5 months old; 50 males and 50 females) were obtained from the Laboratory Animal Center at the Guangzhou University of Chinese Medicine. Rats were housed in an air-conditioned animal house under a normal day/night cycle. All animals were fed a normal rodent chow diet *ad libitum* and had free access to water. Food intake was monitored daily. This study was approved by the Animal Care Committee of Guangzhou University of Chinese Medicine and conducted in accordance with the institutional guide for the care and use of laboratory animals.

### Experimental design

Prior to the experiment, rats were administered tropicamide eye drops to check their lenses under the slit lamp (YZ-5E slit lamp microscope, Suzhou medical apparatus and instruments factory, P.R. China). After one week of adaptable feeding, the rats were divided into six groups. Control rats (group I; *n* = 15) received only 0.1 M citrate buffer, pH 4.5 as vehicle. Other animals in experimental groups were overnight-fasted, then diabetes (type I) was induced by a single intraperitoneal injection of 1 % (W/V) STZ (30 mg/kg body weight) in 0.1 M citrate buffer, pH 4.5 [47]. Three days after the STZ injection, blood was drawn from the tail vein to measure blood glucose levels. Rats with blood glucose levels over 130 mg/dL were selected to develop cataract, then their eyes were examined daily. When the lens opacity reached stage 2 on day 30 (The noncataractous animals were eliminated), the rats were then randomly distributed into five groups (Groups II-VI, *n* = 15/group): DC rats treated with normal saline (group II, model group), DC rats treated with syringic acid eye drops (group III), DC rats treated with gigantol eye drops (group IV), DC rats treated with combination gigantol and syringic acid eye drops (group V), and DC rats treated with 0.053 μg/μL pirenoxine sodium eye drops as suggested by the manufacturer (group VI, positive control group). Drug administration was performed by applying 50 μL of eye drops to each eye, 3 times per day, for 60 consecutive days.

### Slit lamp examination and cataract classification

Eyes were examined daily for 90 days using a slit lamp microscope on dilated pupils. Initiation and progression of lenticular opacity was graded into five categories as follows [28]: stage 0: clear lenses and no vacuoles present; stage 1: vacuoles cover approximately one half of the surface of the anterior pole, forming a subcapsular cataract; stage 2: some vacuoles have disappeared and the cortex exhibits a hazy opacity; stage 3: a hazy cortex remains and dense nuclear opacity is present; and stage 4: a mature cataract is observed as a dense opacity in both cortex and nucleus. The incidence of cataract appearance is expressed as the percentage of total lenses in each group. At the end of 90 day, animals were sacrificed by CO_2_ asphyxiation, and the lenses were dissected by the posterior approach. Then a small incision was made on the posterior side of the eye with the scissors. The lenses were collected by pressing with tweezers against the side of the eye opposite of the incision and stored at −80 °C until further analysis.

### Western blot analysis

Western blot analysis was performed as previously described [48]. Protein samples were prepared by sample homogenization in radio immunoprecipitation assay (RIPA) buffer (1 % Nonidet P40, 0.5 % sodium deoxycholate, 0.1 % sodium dodecyl sulfate-polyacrylamide in PBS) [49]. Equal amounts (20 μg / lane) of protein were subjected to a 10–20 % sodium dodecyl sulfate-polyacrylamide gel electrophoresis (SDS-PAGE) gradient and then transferred to Polyvinylidene-Fluoride membranes (Scientific Research Special, USA). Membranes were blocked for 1 h with 5 % nonfat dry milk in 0.1 % Tween–20 in PBS and then incubated overnight at 4 °C with AKR1B1 antibody (ABGENT, CA, USA) at 1:1000 dilution. Following 3 washes with PBS containing TBST (Tris and Tween Solution Buffer), the membranes were incubated with HRP (horseradish peroxidase)-labeled anti-mouse immunoglobulin G. The specific band was visualized with BeyoECLPlus (Santa Cruz Biotechnology, TX, USA). The density of each band was analyzed using a LAS-1000 UV mini imager (Fuji Film, Tokyo, Japan).

### Real-time reverse transcriptase-PCR

Total RNA was isolated from frozen rat lens samples from each of the 5 groups (group I to V) using Trizol reagent (Invitrogen, CA, USA) as previously described [33, 50]. Lens cDNA was synthesized using the ImProm-II™ Reverse Transcription System kit (Promega Company, Madison, WI, USA) in accordance with the manufacturer’s protocol. The following primers were used: 5’-AGC GGT TTA GGT ACC ATG GGT TTT-3’ and 5’-AGG GTA AGC TTC GAA TTC TCA GGC GCG GAT TTG TTG TGA-3’ for AR,5’GAGACCTTCAACACC CAGCC-3’ and 5’-GCGGGGCATCGGAACCGTCA-3’ for β-actin. Quantitative real-time PCR was performed with Power SYBR Green PCR Master Mix (Applied Biosystems, Norwalk, CT, USA) using a 2400 Real-Time PCR system (Applied Biosystems, Norwalk, CT, USA). The following PCR cycling conditions were used: 95 °C for 10 min, with 40 cycles at 95 °C for 15 s and 60 °C for 1 min. The relative quantities of AR mRNA were automatically assessed by the comparative cycle threshold method and normalized to β-actin mRNA levels as an endogenous control [51].

### Sorbitol assay in the lens

The lens homogenate from each of the five groups (groups I to V) was prepared in PBS (pH 7.4). Sorbitol levels in the lens were measured as previously described [52, 53]. Briefly, a total of 1 mL of extract from each lens was mixed with 2 mL glycine buffer (0.05 M, pH 9.4) containing 2 mM NAD (Nicotinamide adenine dinucleotide) and 0.05 mL sorbitol dehydrogenase (25.6 U/mL) and incubated at room temperature for 60 min. After incubation, fluorescence of the generated NADH was measured at an excitation wavelength of 366 nm and emission wavelength of 452 nm using a spectrofluorometer (F-4500, Hitachi, Japan). Sorbitol levels in each extract were calculated from a calibration curve of D-sorbitol. Sorbitol content in the lens was expressed as μmol/g wet weight.

### Construction of pET28a vector for expression of wild-type AR and mutants

**Fig. 1:**
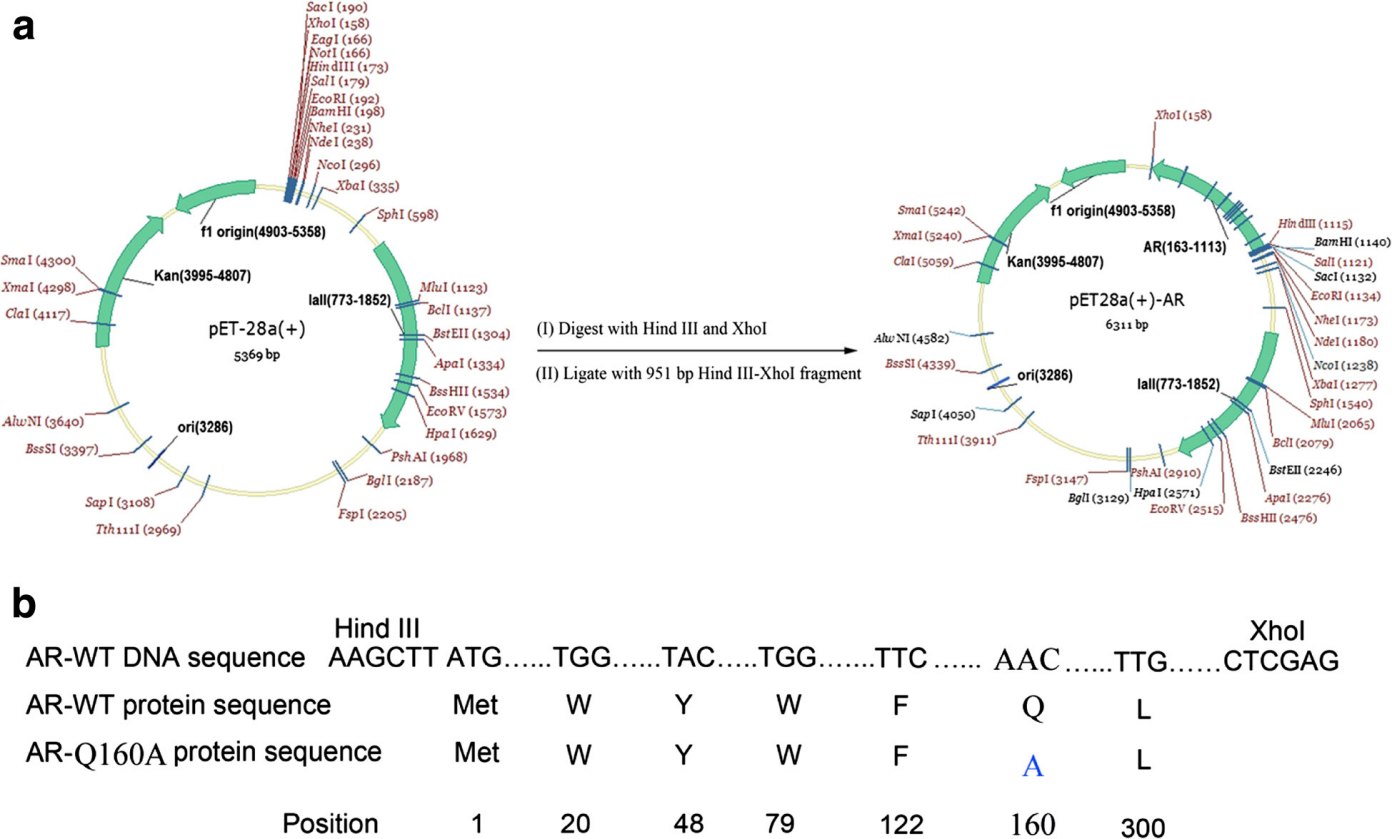
Structure and protein sequence of plasmid pET-28a-AR and its mutants. **a** plasmid map of pET-28a and pET-28a-AR. pET-28a was digested with *HindIII* and *XhoI* and ligated with a 951-bp fragment from either wild-type or mutant AR. **b** Protein sequences of wild-type and mutant AR

The pET28a-AR (Novagen, Germany) plasmid carrying full-length cDNA encoding AR was used as the template, and AR cDNA fragments were specifically amplified using primers containing restriction enzymes *Hin*dIII (TaKaRa, Japan) and *Xho*I (TaKaRa, Japan) digestion sites; after amplification, these fragments were cloned into the pET28a expression vector. The accuracy of the insertion sequence was verified by sequencing. AR Asn160 mutant expression plasmids were constructed using pET28a-AR expression plasmid as template and rapid one-step PCR-medicated site-directed mutagenesis. Asn160 was mutated to alanine (Ala) (Fig. 1). The primer sequences for mutagenesis are listed in Table 2; primers were synthesized by Shanghai Biotechnology Co., Ltd. P.R. China. Experimental procedures were performed according to the instructions provided with the Quick Change XL site-directed mutagenesis kit. Sequencing was performed to verify that no additional mutation (other than the desired Asn160Ala mutation) was introduced into the construct. Sequencing of the recombinant plasmids was carried out by BGI Genomics Co., Ltd, Shenzhen, P.R. China, and this was followed by analysis using the Basic Local Alignment Search Tool (BLAST).

**Table 2:** Primers for human wild-type AR mRNA expression vector and mutant AR-Asn160 expression vector

Primer	Sequence
wild-type AR	*Hin*dIIIF:5’ CCCAAGCTTATGGCAAGCCGTCTCCTG 3’ *Xho*IR:5’ CCGCTCGAGTCAAAACTCTTCATGGAAGGG 3’
mutant AR-Asn160	*Hin*dIIIF:5’ CCCATCCTGGGGTTGGGTACCGCTAAGGGTGGTGGTTCTGGAGGTG3’ *Xho*IR:5’ CTGCCCTGGAGGGGACTTAGCGGTACCTCCCTGGCTATTATCTGAGCTT 3’

### Gene expression and purification

The pET28a-AR and mutant pET28a-AR Asn160 expression vectors were used to transform *E.coli*BL21 (DE3) (Novagen, Germany). *E. coli*BL21 (DE3) were used for expression of wild-type and mutant AR. Bacteria were cultured on LB (Luria-Bertani) plates containing Kanamycin (final concentration 50 μg/mL); incubation was performed overnight at 18 °C with constant shaking at 180 rpm. Transformation of *E. coli*BL21 (DE3) competent cells with blank vector pPELB served as the control. Successfully transformed positive clones were screened. Expression was detected by 12 % sodium dodecyl sulfate polyacrylamide gel electrophoresis.

The bacteria solution was centrifuged at 10,000 rpm for 30 min at 4 °C, mixed with lysis buffer (50 mM NaH_2_PO_4_, 300 mM NaCl, 20 mM imidazole, pH = 8.0), disrupted by ultrasonic treatment after suspension, and centrifuged at 10,000 g, 4 °C for 30 min. The supernatant was transferred to a centrifuge tube, mixed with lysis buffer-treated Ni-NTA beads, and then slowly shaken on ice for 1 h to fully mix the beads and protein. The bead-protein solution was transferred to a chromatographic column, and the beads were allowed to naturally sediment. Next, the beads were washed twice with 8 mL eluent (50 mM NaH_2_PO_4_, 300 mM NaCl, 20 mM imidazole, pH = 8.0). The target protein was eluted into 1.5 mL EP tubes using elution buffer (50 mM NaH_2_PO_4_, 300 Mm NaCl, 250 mM imidazole, pH = 8.0); between four and six elutions were performed, and 1 mL eluent was collected each time. Steps of the procedure involving sedimentation, rinsing, and elution were all performed in a refrigerator. Protein concentration was determined by the Bradford method. Sample purity was determined by 12 % sodium dodecyl sulfate polyacrylamide gel electrophoresis. Purified protein plus 15 % glycerol (Amresco, USA) was stored at −80 °C.

### Measurement of AR activity

Enzyme activity was determined according to a previously described method [54]. The decrease in NADPH absorbance per minute in response to different concentrations of combination treatment of gigantol and SA was determined by plotting the inhibitory rate of each sample for wild-type AR (wt-AR) compared to mutant AR Asn160 (Q160A).

### Statistical analysis

Statistical analyses and data processing were performed using SPSS 22 software. *P* < 0.05 was considered statistically significant.

## Results

### Gigantol and syringic acid synergize to enhance viability of HLECs

Compared to either the normal control group or the osmolarity control group, HLECs treated with high glucose (50 mM) became swollen and exhibited cavitation and formation of small particles when viewed under an inverted microscope. The decrease in cell density and the increased numbers of floating cells suggested that high glucose inhibits HLECs proliferation. Treatment of these cells with various concentrations of gigantol and SA, either alone or in combination, restored HLECs morphology back to normal. MTT assay showed that HLECs viability in the model group was significantly decreased as compared to any of the other treatment groups (Table 3; *P <* 0*.*01). There was no difference in viability between the normal control group and the osmolarity control group (*P* > 0.05), suggesting that osmolarity had no significant effect on HLECs viability.

**Table 3:** Cell viability (%) of HLECs after treatment with gigantol, syringic acid, and the combination in the presence of high glucose

gigantol (μg/mL)	syringic acid (μg/mL)				
	0	0.125	0.625	1.25	2.5
0	28.23 ± 1.12^*^	32.62 ± 1.56	36.21 ± 2.18	41.52 ± 1.53	47.72 ± 1.49
0.1	35.33 ± 1.87	68.02 ± 1.27	69.01 ± 1.79	71.09 ± 1.94	73.99 ± 1.35
0.5	39.55 ± 1.25	69.77 ± 2.22	75.05 ± 2.15	76.26 ± 1.28	78.96 ± 1.57
1	44.71 ± 2.51	71.16 ± 1.61	73.66 ± 1.24	82.51 ± 1.53	83.02 ± 1.82
2	50.65 ± 1.47	72.76 ± 1.59	74.13 ± 1.27	83.43 ± 1.56	89.16 ± 1.32

Cells were incubated with various concentrations of gigantol, SA and their combination in presence of high glucose (50 mM, containing medium) for 72 h. The cell viability of normal group (5.56 mM glucose), osmolarity control group and model group were 100 ± 0.89 %, 98.33 ± 0.24 % and 28.23 ± 1.12 %, respectively. Cells were treated for 72 h, respectively. independent experiments. Data were expressed as mean ± SD (*n* = 3), ^*^ *P* < 0.01 vs all other groups

Using the Jin’s formula [37, 38], we found that gigantol and SA in combination yielded synergism across a wide range of concentrations; in total, nine combination treatments showed synergism (q ≥ 1.15) while the remaining combinations were additive (Table 4). The largest degree of synergism was observed when gigantol and SA were administered at a ratio of 1:1.25 (w/w) (*P <* 0*.*01). As such, concentrations of 1 μg/mL gigantol and 1.25 μg/mL syringic acid were chosen for further investigation for combined effects and mechanisms.

**Table 4:** Synergistic effect of gigantol combined with SA on high glucose-induced HLECs analyzed by Jin’s formula (q value listed in the table)

gigantol (μg/mL)	syringic acid (μg/mL)			
	0.125	0.625	1.25	2.5
0.1	1.21^a*^	1.17^a^	1.14	1.10
0.5	1.18^a^	1.22^a*^	1.18^a^	1.15^a^
1	1.13	1.14	1.26^a*^	1.17^a^
2	1.09	1.06	1.12	1.20^a*^

According to Jin’s formula, q < 0.85 indicates antagonism, 0.85 ≤ q < 1.15 indicates additive effects, and q ≥ 1.15 indicates synergism (^a^). Synergism indicates that the effect of a mixture exceeds that expected from the individual components and additive effects (non-interaction) mean that the combined effect is equal to the expectation. The largest grades synergism efficiency when combination ratio of gigantol and SA was 1:1.25, ^*^ *P* < 0.05 vs the other combination ratio groups

### Gigantol and syringic acid synergize to inhibit DC lens opacification in STZ-induced rat model *in vivo*

**Fig. 2:**
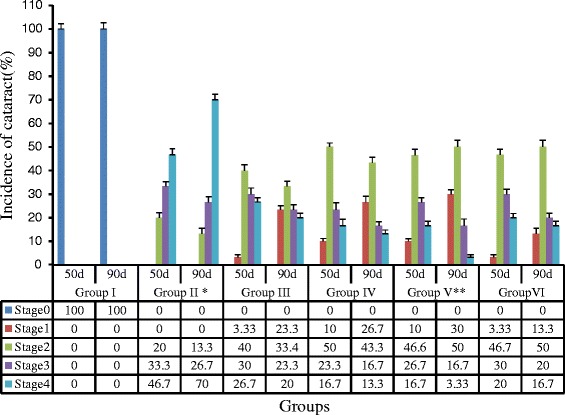
Cataract progression in different groups as observed by slit lamp microscope. The results are expressed as mean ± SD (*n* = 15). Compared with the model group (Group II), turbidity in the pirenoxine group (GroupVI) and all 3 treatment groups (Group III, IV, V) was significantly reduced, ^*^ *P* < 0.01, vs all other groups. Optimal effects were observed in the combination (Group V); ^**^ *P* < 0.05, vs group III, IV

**Fig. 3:**
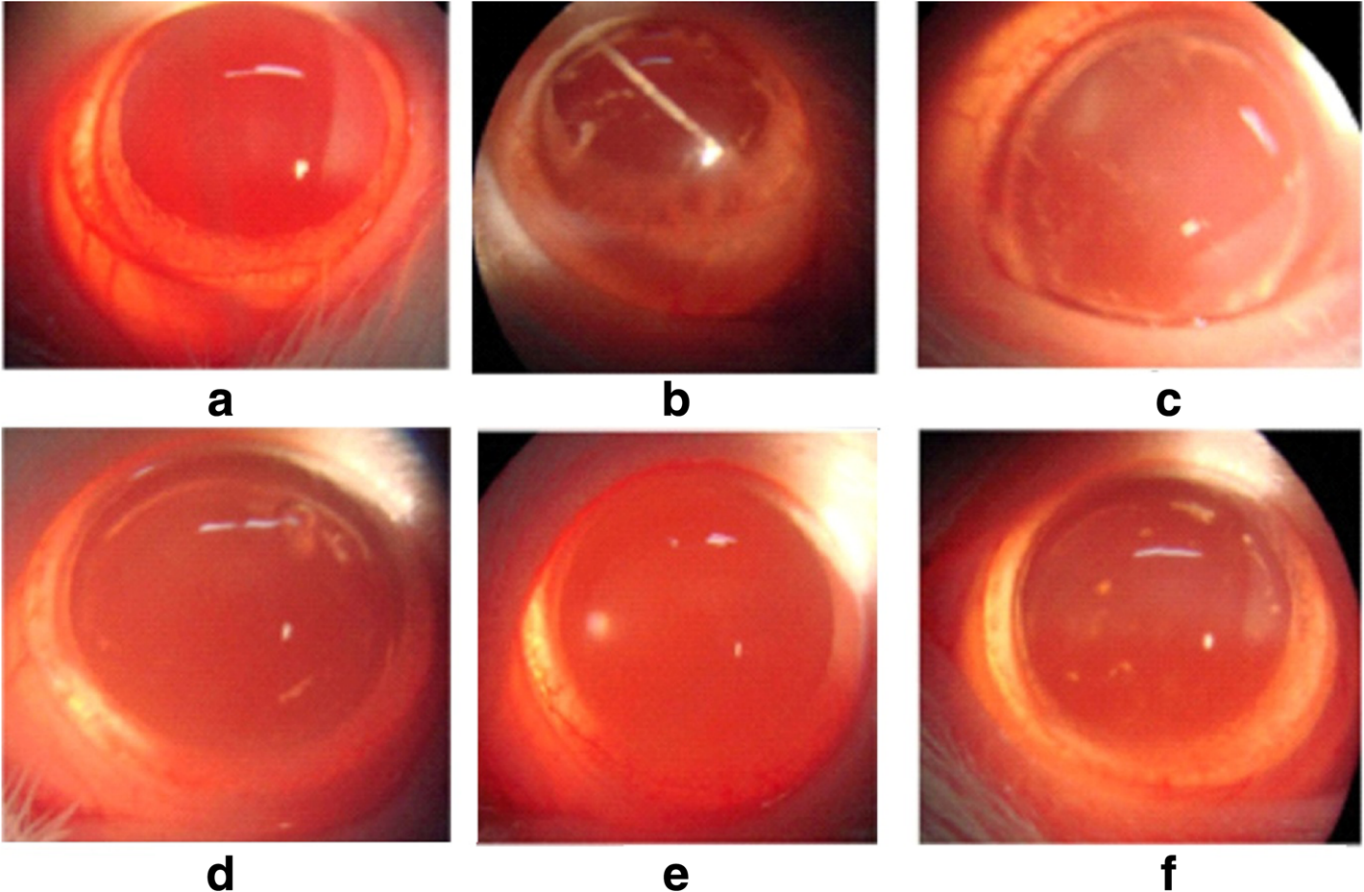
Inhibitory effect of gigantol and SA eye drops on STZ-induced diabetic cataracts in Wistar rats. Representative photographs of lens from each group at the end of 60 days eye drop treatment. **a** normal control (group I); **b** diabetic cataracts untreated (group II); **f** diabetic cataracts treated with pirenoxine sodium eye drops each time (group VI); **c**, **d**, and **e** diabetic cataracts treated with SA, gigantol, or the combination of gigantol and SA, respectively (group III, IV and V)

We next examined the effect of gigantol and SA eye drops on cataract formation in the STZ-induced rat model. Cataract scores in each of the experimental groups following 60 days of treatment are summarized in Fig. 2, and representative images of lenses from each group are shown in Fig. 3. The lenses of normal control rats (Group I) appeared to be clear and free of opacities throughout the experimental period. This was in stark contrast to clearly visible cataracts observed in the diabetic model group (Group II). Cataract development was inhibited in the rats treated with gigantol, syringic acid, or pirenoxine (Group III, IV, VI). Importantly, there was a significant difference in cataract progression in the model group compared to each of these three treatment groups (Groups III, IV, VI; *P* < 0.01). Moreover, the combined administration of gigantol and SA (Group V) was more effective than either agent alone (*p* < 0.05).

### Gigantol and syringic acid inhibit AR activity in cultured HLECs

We next examined the effect of gigantol and SA on AR activity in cultured HLECs. HLECs cultured in the presence of 5.56 mM glucose exhibited AR activity of 5.35 mU · min-1 · μg-1 protein. Following 72 h of incubation with 50 mM glucose, AR activity was dramatically increased (*P* < 0.01) to 15.34 mU · min-1 · μg-1 protein, suggesting that high glucose can significantly activate AR. Compared to the high glucose group, AR activity in the gigantol, syringic acid, and combination groups was significantly decreased (*P* < 0.01), suggesting that treatment with these compounds inhibits AR activation induced by high glucose in a dose-dependent manner (Table 5).

**Table 5:** Inhibition efficiency of drug alone and drug combination on AR activation (mU · min^−1^ · μg^−1^ protein) of HLECs induced by high glucose

gigantol (μg/mL)	syringic acid (μg/mL)				
	0	0.125	0.625	1.25	2.5
0	16.34 ± 0.71^*^	13.11 ± 0.13	12.23 ± 0.68	11.21 ± 0.37	10.27 ± 0.29
0.1	12.98 ± 0.32	10.46 ± 0.23	9.79 ± 0.33	8.86 ± 0.19	8.45 ± 0.58
0.5	11.02 ± 0.63	10.06 ± 0.26	9.51 ± 0.15	8.62 ± 0.47	8.05 ± 0.36
1	10.52 ± 0.42	9.73 ± 0.54	9.06 ± 0.27	8.03 ± 0.36	7.24 ± 0.83
2	10.01 ± 0.76	9.33 ± 0.45	7.42 ± 0.52	6.79 ± 0.23	6.25 ± 0.37

The AR activity in HLEC cultured in the normal group (5.56 mM glucose alone) was 5.35 mU · min^−1^ · μg^−1^ protein. The results given are for HLEC incubated with 50 mM glucose with the indicated additions, respectively. Data were expressed as mean ± SD (*n* = 3). ^*^ *P* < 0.05 vs all other groups

### Gigantol and syringic acid attenuate STZ-induced AR expression in the lens

**Fig. 4:**
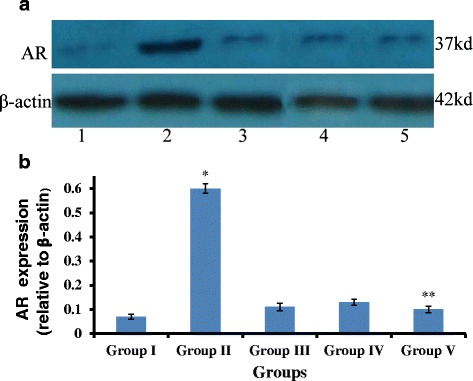
AR expression in the lens after treatment with gigantol, syringic acid, and the combination. **a** Representative image of Western blot, lanes 1 to 5: Group I to Group V. β-actin was used as the internal control. **b** AR expression levels after normalization for β-actin. The values are mean ± SD (*n* = 20), *p < 0.01, vs Group I, III, IV, V; **p < 0.05, vs Group III, IV

The effect of gigantol and SA on AR activity prompted us to examine the effect of these compounds on its protein expression. We performed Western blot analysis for AR expression in the lens from the various treatment groups (Fig. 4). AR expression in animals with diabetic cataracts (Group II) was approximately 6-fold higher than in the normal control group (*p* < 0.01). This increase was significantly inhibited by gigantol and SA, and, importantly, the combined treatment of gigantol and SA performed better than either agent alone (*p* < 0.05). These findings indicate that combination gigantol/syringic acid significantly attenuates STZ-induced AR expression in vivo, which may, at least in part, explain the synergistic effect of combination treatment.

### Gigantol and syringic acid attenuate STZ-induced AR mRNA expression in the lens

**Fig. 5:**
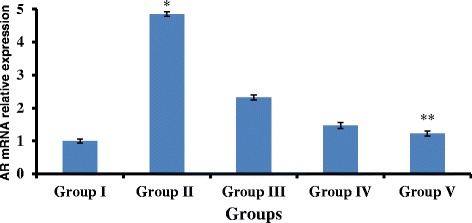
AR mRNA expression after treatment with gigantol, syringic acid, and the combination. The expression of AR mRNA is normalized using the housekeeper gene (*β*-actin), which is evenly expressed in rat lens cells. Compared with the normal control group, mRNA levels of the AR gene increased 4.85-fold in Group II and 1.23-fold in Group V; *p < 0.01 vs Group I, Group III, Group IV and Group V. **p < 0.05 vs Group III and IV

Since we found that gigantol and SA modified AR protein expression, we next examined whether these compounds regulated AR at the mRNA level as well. We performed real-time RT-PCR and found low level of AR mRNA expression in control rats (Group I). AR mRNA transcripts were significantly elevated in the diabetic model group, showing a 4.85-fold increase in AR mRNA expression in STZ-induced rats (Fig. 5). This STZ-induced increase was attenuated by gigantol and syringic acid (*p* < 0.01). Importantly, combined administration (group V) was more effective than either compound alone (*p* < 0.01), indicating that gigantol and syringic acid synergistically inhibit STZ-mediated increase in AR mRNA expression.

### Gigantol and syringic acid decrease sorbitol accumulation in DC lenses

**Fig. 6:**
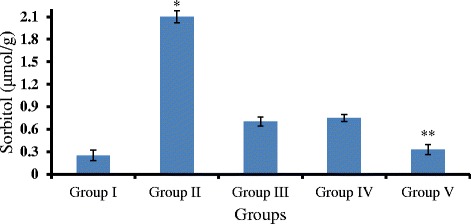
Sorbitol levels in the lens after treatment with gigantol, syringic acid, and the combination. The sorbitol level in lens in DC rats is significantly higher than levels in control rats (*p* < 0.01). Gigantol, syringic acid, and the combination reduced STZ-induced lenticular sorbitol accumulation. Sorbitol levels in lenses treated with the combination are lower than in the gigantol or syringic acid groups alone (*p* < 0.05)

We found elevated sorbitol levels in lenses from DC rats compared to control rats (*p* < 0.01). Treatment with either gigantol or SA effectively attenuated this STZ-mediated increase. Moreover, combined treatment with gigantol and SA performed better than either agent alone (*p* < 0.05; Fig. 6), suggesting that these compounds synergize to decrease sorbitol accumulation in the diabetic lens in vivo.

### Generation and expression of wild-type AR and Asn160Ala mutant

Using recombinant plasmid pET28a-AR as template, we performed site-directed mutagenesis to generate the AR Asn160Ala mutant. As expected, a 1 kb band was amplified. This amplified product was digested with *Dpn*I and transformed into *E. coli* BL21 (DE3). Positive clones were screened and sequenced. Following the sequencing and assembly of sequencing data, the BLAST result showed that the AR mutant with Asn160 was changed to Ala and was generated to test the binding sites predicted by docking simulation.

**Fig. 7:**
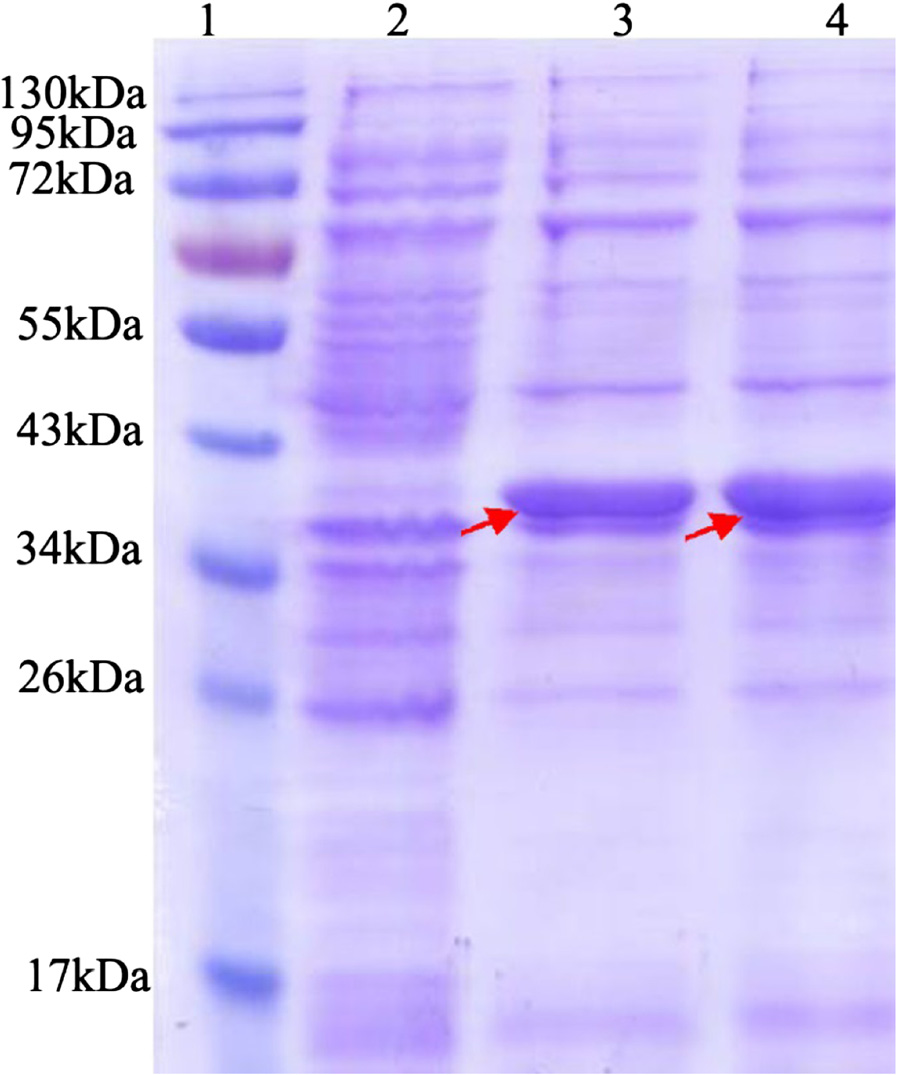
Expression of wild-type and mutant AR (sodium dodecyl sulfate polyacrylamide gel electrophoresis). BL21 (DE3)-pET28b-AR and the mutant were induced by 0.5 mM IPTG at 18 °C and then purified. Sampling volume was 8 μg per well. 2 proteins were successfully purified. AR protein molecular mass was 34.8 kDa, and was 38 kDa after being fused to His. 1: Marker; 2: blank control; 3: wild-type AR; 4: AR N160A. The arrow indicates the target protein

Differential expression was analyzed by sodium dodecyl sulfate polyacrylamide gel electrophoresis. Wt-AR and mutant AR were expressed at similar levels (Fig. 7). As expected, both wild-type and mutant proteins ran at approximately 36000 Da. Protein concentration was determined by the Bradford assay. The concentration of wt-AR and the mutant Q160A was 1.85 and 7.93 mg/mL, respectively.

### Asn160 is a key residue within AR mediating the inhibitory effect of gigantol and syringic acid

**Fig. 8:**
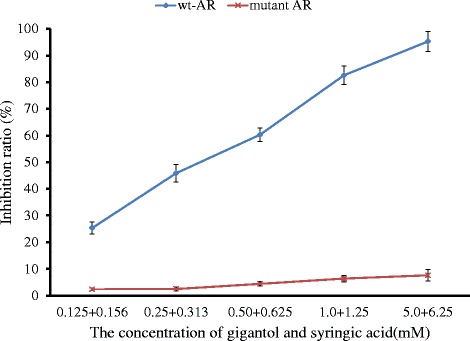
Wild-type AR (wt-AR) and the mutant AR (Q160A) activity in the presence of different concentrations of gigantol and syringic acid. Data are expressed as mean ± SD (*n* = 3)

Combined treatment of gigantol and SA inhibited wt-AR in a dose-dependent manner, and the inhibition ratio of combination treatment was consistent with our previous work [34]. Furthermore, we found that the inhibitory effect increased with increasing concentrations of both compounds. In contrast to wt-AR, the inhibition ratio of Q160A mutant AR in the presence of similar concentrations of gigantol and SA was far lower. In fact, gigantol and SA were very poor inhibitors of mutant AR activity (Fig. 8). These data suggest that Asn160 is a key residue within AR mediating the inhibitory effects of gigantol and SA.

## Discussion

The development of cataracts in patients suffering from diabetes can lead to blindness at late stages of the disease. The polyol pathway, advanced glycation end products (AGEs), and oxidative stress have all been implicated in the development of DC [55–58]. Lens epithelial cells (LECs) are damaged in diabetic cataractogenesis, which can be induced by ultraviolet radiation, oxidative stress, and hyperglycemia [59–61]. Apoptosis of LECs occurs during cataract formation, and, as such, inhibiting apoptosis can prevent or delay this process [60, 62]. AR catalyzes the conversion of glucose to sorbitol via the polyol pathway, a process involved in diabetic cataract formation [63–67]. Extensive research has demonstrated that the AR pathway is the initiating step in diabetic cataract formation. Intracellular accumulation of sorbitol leads to osmotic changes resulting in hydropic lens fibers that degenerate and form sugar cataracts [68, 69]. There are several factors leading to the accumulation of sorbitol in the lens. The production of sorbitol by AR occurs more rapidly than its conversion to fructose by sorbitol dehydrogenase [70–73]. In addition, sorbitol cannot penetrate the cell membrane by simple diffusion. As a result, increased accumulation of sorbitol creates a hyperosmotic effect that leads to an infusion of fluid to countervail the osmotic gradient [68]. Studies have shown that osmotic stress in the lens caused by sorbitol accumulation also induces LEC apoptosis, leading to the development of cataract [74–77].

The Dendrobium species (Orchidaceae), locally known as ‘Shihu’ or ‘Huangcao’, have long been used in traditional Chinese medicine for their antipyretic, eye-benefiting, immunomodulatory, and anti-aging effects [78, 79]. Our previous studies performed in the galactose- and hydrogen peroxide-induced DC animal models have demonstrated that the combination of gigantol and syringic acid inhibits AR activity and prevents cataract formation more effectively than either compound alone [34]. Molecular docking analysis predicted that the synergistic binding site between AR and combined gigantol and SA administration was the amino acid residue Asn160 within AR [34]. In the present study, we demonstrate that gigantol and syringic acid synergistically inhibit AR activity and protect against cataract formation both in vitro and in vivo. Importantly, this synergistic effect was observed in multiple diabetic cataract models, including the high glucose-, STZ-, and D-galactose-induced models [34].

Galactosemic animal models are widely used to study sugar-induced complications. This is because galactose can rapidly produce cataracts, and animal survival in this model is typically better due to less severe systemic metabolic changes. As such, this animal model is often favored over the diabetic model, particularly for initial screening of new investigational agents [80]. Therefore, we used this model in our previous work to initially screen active ingredients from Dendrobium [32–35]. While appropriate as a starting point, galactose- and hydrogen peroxide-induced DC models are not exact representations of human DC. Despite some similarities, including activation of AR, polyol accumulation, and oxidative stress [80–85], we transitioned to using the STZ-induced DC model in the study presented here.

In an effort to understand the synergistic mechanism of gigantol and syringic acid, we treated STZ-induced rats with a combination of both compounds. We found that combined gigantol and SA treatment inhibited HLECs apoptosis and lens opacification. To better understand the underlying synergistic effect, we examined AR activity, expression level of AR, and sorbitol accumulation in the lens. We found that treatment with gigantol and/or syringic acid decreased AR activity and expression, particularly in the combination group. Furthermore, a significant decrease in the accumulation of sorbitol was observed when both compounds were simultaneously administered, which correlated with decreased HLECs apoptosis. AR downregulation by gigantol and/or syringic acid might explain their synergistic anti-cataract effect in the lens.

We next examined the importance of Asn160 in AR for its inhibition by gigantol and SA. Enzyme kinetics showed that the Q160A mutant was not inhibited by gigantol and SA to the same extent as the wild-type protein. These findings confirmed that Asn160 is a key residue within AR mediating the inhibitory effects of gigantol and SA, and this information was not previously reported [86–92]. The bond between gigantol, SA, and AR is very stable. Combination treatment and AR form their collaborative pharmacophore. We found that the hydrogen bond receptors were the oxygen of 3-methoxyl in the benzene ring of SA and the 5-methoxyl group in the benzene ring of gigantol; the hydrogen bond donors were the oxygen of the 3-methoxyl group in the benzene ring of syringic acid. Thus, gigantol and syringic acid provide synergistic protection against the development of DC through inhibition of AR activity.

## Conclusion

In conclusion, combined treatment of gigantol and syringic acid synergize to significantly protect against DC formation and, as such, may represent a promising therapeutic option. The synergistic effect might be due to decreased AR expression and activity. These results add to the growing body of evidence supporting a therapeutic role for gigantol and SA in preventing cataract formation in patients with diabetes.

## Abbreviations

Ala, alanine; AR, aldose reductase; ARI, aldose reductase inhibitors; BLAST, basic local alignment search tool; cDNA, complementary DNA; Da, Dalton; DC, diabetic cataracts; DMEM, Dulbecco’s modified Eagle’s medium; DMSO, dimethyl sulfoxide; FBS, fetal bovine serum; HLECs, human lens epithelial cells; MD, molecular docking; MTT, 3-(4,5-dimethyl-2-thiazolyl)-2,5-diphenyl-2-H-tetrazolium bromide; NAD, nicotinamide adenine dinucleotide; NADPH, reduced nicotinamide adenine dinucleotide phosphate; OD, optical density; PBS, phosphate buffer saline; PCR, polymerase chain reaction; PDB, protein data bank; Q160A, Ala substitution at Asn 160; RIPA, radio immunoprecipitation assay; SA, syringic acid; SDS-PAGE, sodium dodecyl sulfate-polyacrylamide gel electrophoresis; STZ, streptozotocin; wt-AR, wild-type AR
